# The Role of Attentional Priority and Saliency in Determining Capacity Limits in Enumeration and Visual Working Memory

**DOI:** 10.1371/journal.pone.0029296

**Published:** 2011-12-16

**Authors:** David Melcher, Manuela Piazza

**Affiliations:** 1 Center for Mind/Brain Sciences (CIMeC), University of Trento, Rovereto, Italy; 2 Department of Cognitive Sciences, University of Trento, Rovereto, Italy; 3 Institut National de la Santé et de la Recherche Médicale (INSERM) Cognitive Neuroimaging Unit, NeuroSpin Center, CEA Saclay, Gif-sur-Yvette, France; Istituto di Neuroscienze, Italy

## Abstract

Many common tasks require us to individuate in parallel two or more objects out of a complex scene. Although the mechanisms underlying our abilities to count the number of items, remember the visual properties of objects and to make saccadic eye movements towards targets have been studied separately, each of these tasks require selection of individual objects and shows a capacity limit. Here we show that a common factor—salience—determines the capacity limit in the various tasks. We manipulated bottom-up salience (visual contrast) and top-down salience (task relevance) in enumeration and visual memory tasks. As one item became increasingly salient, the subitizing range was reduced and memory performance for all other less-salient items was decreased. Overall, the pattern of results suggests that our abilities to enumerate and remember small groups of stimuli are grounded in an attentional priority or salience map which represents the location of important items.

## Introduction

Many common tasks require us to process in parallel multiple objects in a complex scene. However, in order to make specific decisions based on the identity, location, and functional properties of individual objects, it is necessary to select and process individual objects. We call this last process “individuation”, to emphasize that by such mechanism items are perceived as specific individuals. It has long been known that the number of items that can be individuated in a single glance is limited [1]. The mechanisms underlying this fundamental limit in human cognition remain a topic of considerable debate [2]-[4].

This capacity-limited ability is clearly evident when people are engaged in an enumeration task: they can assess the exact number of items in a visual array without effort, being fast and extremely accurate when the items are few, up to 3 or 4 (a phenomenon called “subitizing”). However, when the number of items exceeds 3–4, enumeration becomes slow and it relies on the coordination of several visual-spatial and symbolic operations (“counting”). Alternatively, if counting is made impossible, for example by short exposure to stimuli, subjects rely on a number estimation system, which is slow, imprecise, and governed by Weber's law. According to the “numerical” hypothesis [5], subitizing reflects this basic ability to estimate the number of objects in a collection and thus is indistinguishable from estimation. According to a “perceptual” account, subitizing differs from both estimation and counting in many respects [6]–[8], perhaps reflecting a particular feature of the visual system that allows parallel individuation of a limited number of multiple objects.

In addition to simply keeping track of the presence of an object, as we do in enumeration, we might also want to encode its visual properties in order to compare it to other objects or to find it later. Like enumeration, visual working memory (VWM) shows strict upper limits of around 3–4 items [9], although the lower limit in capacity varies depending on the participants and task parameters [2],[10]–[12]. There are a number of theories regarding the underlying mechanisms that yield capacity limits, including “slot” models, which like the perceptual hypothesis described above posit a fixed number of objects that can be stored [4], [9], and “resource” models [2], [10] based on a fixed resource which is divided between objects based on the complexity of all items to be encoded.

Both VWM and enumeration share a similar capacity, and indeed individual differences in VWM and subitizing range are correlated [13], [14]. These two tasks, both requiring individuation, also interfere with each other [13], [14], suggesting a common mechanism underlying individuation in both tasks. The question remains, however, of what is the common factor between visual working memory and enumeration. Answering this question requires us to tackle the fundamental issue of why we have capacity limits.

One potential clue for answering the question of why we have capacity limits comes from studies of multiple object tracking, where capacity limits of around 3–4 items are also found. Drew and Vogel [15] found that the ability to initially individuate/select items, as measured by individual differences in the N2pc component of the EEG signal, predicted subsequent performance in the multiple object tracking paradigm [15]. These findings suggest that the individuation/selection step forms a first bottleneck which serves as an upper limit on the ability to track multiple items or maintain them in memory. Similarly, Wood and colleagues have argued that object individuation and tracking is part of the “core architecture” of VWM [16].

In line with these recent studies, we begin with the idea that capacity limits are determined, at least in part, by a first step of competitive interactions based on bottom-up saliency and task relevance. We suggest that this first stage is a sensorimotor representation involved in the individuation of items, a *Map of Attentional Priority and Saliency* (henceforth, “MAPS”), which keeps track of the location of salient items in the scene. The idea of a master saliency map comes from single-cell neurophysiology [17]–[20] and computational modeling [21],[22]. In neurophysiology, saliency maps are used to describe the finding that neurons show increased firing to stimuli which “pop out” from the background items (based on “bottom-up” factors such as color, visual contrast, size and movement) or are behaviorally relevant [17]–[20]. Neurons in the lateral intraparietal (LIP) area, for example, respond based on whether or not the item in the receptive field is salient. The relative saliency of items depends on competition between the various items, such that if one item is particularly salient compared to the other items it can become the only item strongly represented in the map [23]. Regarding these saliency maps, it is interesting to note that there is a suggestive overlap between the areas in posterior parietal cortex which have been implicated in enumeration [24], visual-spatial working memory [25],[26] and sensorimotor saliency [17].

Computational models of saliency maps are winner-take-all models of visual attention. The saliency maps proposed by Koch and Ullman [21] and Itti et al [22] accounted for the integration of "bottom-up" topographic information from feature extraction processes (such as visual contrast, size and movement), and the selection of one most salient area. We use the terminology MAPS here to emphasize the role of both bottom-up salience and task relevance in determining the “attentional priority” of each item [27]. For example, Standage and colleagues [28] developed a model in which top-down factors such as task relevance are also taken into account and in which several areas may achieve equal priority in the map. One key prediction of models based on winner-take-all selection is the existence of competitive interactions, such as lateral inhibition, between items [21], [22], [29], [30]. When this lateral inhibition between items is included in the model, the relative salience/priority of each item can influence how many items are represented in total, such that even a small difference in salience can cause one item to dominate the others [31]. This idea is also supported by clinical studies of neurological patients who show difficulty in individuating a single target item in the presence of highly salient distractors [32].

The second stage, in a working memory task, involves processes which maintain these spatial representations and link them to activity in other areas of the brain which process attributes beyond the scope of posterior parietal cortex. While the first stage provides an initial limit for capacity (based on the competitive interactions between neurons), the second stage helps to explain the wide differences in working memory capacity estimates due to task and individual differences. The frontal-parietal working memory circuit, involving areas such as prefrontal cortex, are likely candidates for maintenance of representations in the map [33]. In addition, many tasks require complex details that would require the activity of object-processing areas in temporal cortex. For example, a color-change task might be possible based on the selectivity of parietal cortex neurons alone [34], while a change in a complex shape might require recruiting areas in temporal cortex [35] and, thus, lead to a reduced capacity in terms of number of items. Likewise, increasing the maintenance requirement, such as by increasing the temporal delay between the memory set and the test, should lead to a decrease in memory capacity estimates [36].

This two-stage theory of capacity limits, grounded in the activity of saliency maps, leads to some specific and testable predictions. In particular, there are three predictions following from our hypothesis which have not previously been addressed. First, we can predict that increasing the relative salience of one item compared to the others should cause it to dominate the attentional priority representation [31], [37]. This leads to the previously-untested prediction that introducing unequal saliency in these tasks would *decrease* overall capacity. This hypothesis is confirmed here in two experiments using different stimulus types.

Second, individual performance in enumeration and VWM tasks should be related based on the shared initial stage of individuation [14]. However, as tasks become more different, such that spatiotemporal individuation itself becomes less important, then performance on the two tasks should diverge. For example, enumeration and VWM performance should be similar if the extra computational resources, such as memory maintenance, are reduced. This hypothesis can be easily tested by increasing the stimulus complexity or the maintenance requirements of the VWM task. In the case of stimulus complexity, using highly complex targets in the VWM task can reduce capacity to only one item, which is consistent with our hypothesis but also eliminates the ability to test individual differences due to a floor effect. We included three different stimulus types in these experiments: oriented gratings (Gabor patches), oriented bars and colored squares. To further test the role of the second stage of visual working memory we manipulated the maintenance requirement by using the post-cue method in a final experiment [36]. Cueing the location of the target immediately after the disappearance of the item should dramatically reduce the need to maintain multiple items in memory. Instead, performance should be primarily limited by the capacity to individuate items and not to keep them in working memory. Our hypothesis was that the capacity limit for each participant in the enumeration and visual working memory task should be highly similar when we minimized memory demands [14]. In contrast, we expected that capacity estimates should diverge when the stimulus was more complex or when the delay increased, since both of these manipulations increase the importance of the memory component of the VWM task.

Third, we predicted that any factor that influences the salience of items (task relevance, visual salience, reward and motivation, etc…) should be combined into a common neural “currency” of salience [17]. This leads to specific predictions about how top-down and bottom-up salience should be combined which we confirmed in the third experiment.

## Experiment 1: Capacity Limits for Individuation and Visual Working Memory

If individuation is grounded in a sensorimotor saliency map of attentional priority, then would expect that manipulating the salience of one item, through either top-down or bottom-up factors, would result in competitive interactions between the “peaks” of the map [31]. This competition would lead the salient item to dominate the map, reducing the overall capacity for individuation. This allows us to make the specific prediction that the *relative salience* of the items should set the overall capacity estimate. In the case of an extremely salient item, then capacity should be reduced to a single item. When all items are equally salient, then the capacity limit should be highest, while intermediate salience of one item should lead to a capacity estimate between one and the maximum capacity. While the total capacity (measured by Cowan's K in the case of the VWM task) should be determined by the relative salience of the items, this capacity estimate could remain relatively constant across set-size.

We can predict that under high levels of saliency inequality, then individuation should converge to only a single item (K = 1). Indeed, we argue that it is critical to be able to converge to a single item. Attentional priority and salience serve to guide action, and many actions (such as saccadic eye movements or grasping with the hand) are targeted to a single item. For example, motor interference occurs when multiple potential targets compete for behavioral responses [38].

Recent evidence for the role of saliency in individuation comes from a recent study on visual memory for icons in a map [39]. A post-hoc analysis of memory performance in that study revealed that the visual saliency of the visual icons—as measured by the Itti et al. [22] computational model—predicted working memory performance. Thus, we predict that even task-irrelevant differences in bottom-up visual saliency should influence which items are individuated in the two tasks.

To date, research on the capacity limits in the domains of enumeration and in visual working memory has remained largely separate (although see [14]). Similarly, studies of the neural underpinnings of enumeration, visual working memory and salience have taken place in parallel. However, as argued by Dehaene and colleagues, the parietal cortex is a common nexus for visual-spatial representation and number [40] and is a likely candidate for the location of what Gottlieb [17] has called a “master map” (or network of maps) which underlies the individuation of salient items in a complex scene. Thus, we predict that if the two tasks share a common individuation stage, driven by low-level salience, then the influence of saliency should be similar in the two tasks.

### Methods

#### Subjects

Six adult subjects gave informed consent to participating in the experiment. All experiments were approved for human subjects by the ethics committee of the University of Trento.

#### Stimuli

The stimuli used in the VWM, enumeration and orientation tasks were Gabor stimuli (oriented contrast gratings windowed by a Gaussian function), displayed against a mean gray background, with a fixation point in black near the center of the screen. Each Gabor stimulus subtended 1° (except when its size was increased to 2° as part of the saliency manipulation) in visual angle and was located in one of 16 positions in a 4×4 (8° x 8°) grid centered at fixation. In the baseline condition, Gabor stimuli were shown at 30% of full contrast (or up to 100% contrast in the high salience condition) against a mean gray background on a monitor with a mean luminance of 16.4 cd/m^2^. Pilot testing revealed that nine stimuli at baseline contrast were clearly visible and could be accurately counted given sufficient time.

#### Procedure

**Figure 1:**
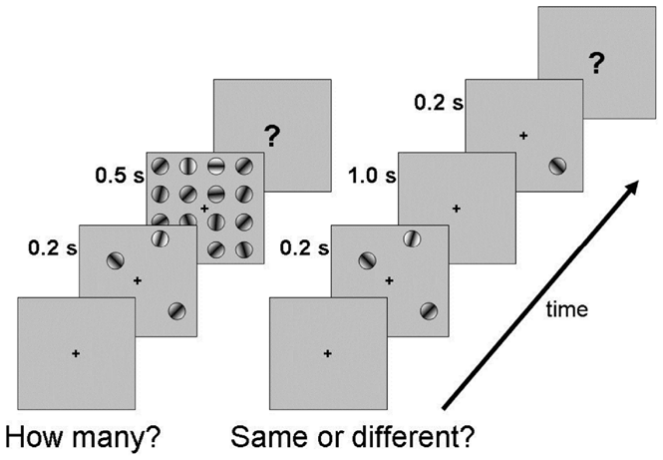
Illustration of experimental methods in the two baseline conditions. On each trial, a test set of stimuli (1–9 items) was briefly presented, either for an enumeration task (left panel) or a visual working memory task (right panel). See Methods for exact parameters of the Gabor stimuli and display.

A fixation point was maintained at the center of the screen throughout each block of trials. Trials were started by a button press, and then after a delay (500 – 700 ms) the first stimulus frame was shown. There were two main measures of *individuation*: enumeration and visual working memory (Figure 1). In both tasks, the initial stimulus set was shown for 200 ms. This brief duration discouraged subjects from making saccadic eye movements to scan the individual items. In the enumeration task (Figure 1, left panel), the stimulus set contained from 1 to 9 Gabor stimuli and was immediately followed by a mask (500 ms) in order to prevent sequentially counting of the items. In the enumeration task, the orientation of the individual Gabors was chosen randomly from all possible orientations.

In the VWM trials there were 1 to 4 Gabor stimuli in the first stimulus set (called the “memory set”) shown for 200 ms. The orientation of each Gabor in the memory set was one of eight possible orientations (+-10, 20, 30 or 40 degrees from the vertical). After the 200 ms display of the memory set, there was a blank delay of 1000 ms. Then one probe stimulus (“test”) was shown for 200 ms followed by a blank screen. On “same” trials, the test Gabor was identical to the Gabor at the same location in the memory set. On “different” trials, the orientation of the test stimulus was mirror-reversed across the vertical. So, for example, on a “different” trial a +20° oriented memory item would be flipped to −20 ° orientation. The change in orientation, therefore, ranged from 20° to 80° in the various trials, making the change “categorical” since the change was an order of magnitude above orientation thresholds.

**Figure 2:**
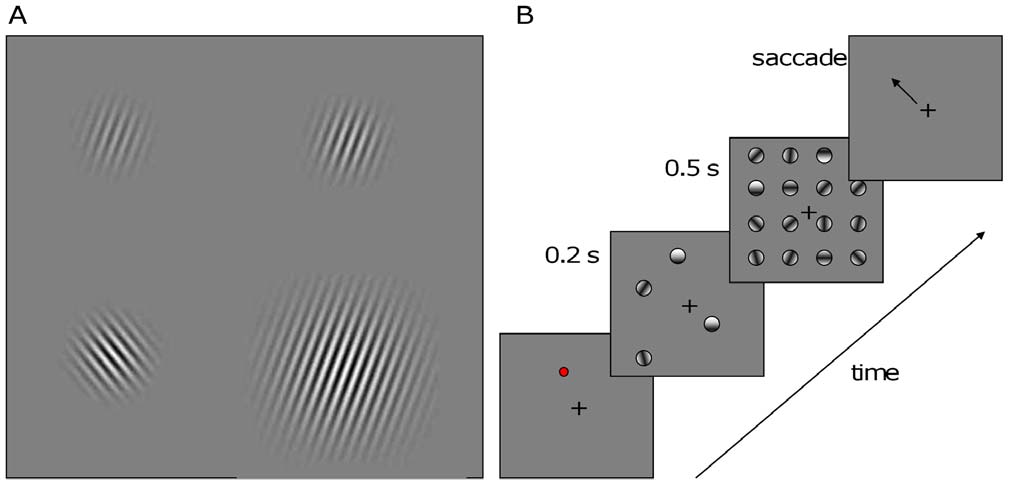
The saliency of one item was manipulated either in a bottom-up fashion by increasing its contrast and/or size (left panel) or by placing one item at the memorized location for a delayed saccade task (right panel).

In addition to the baseline conditions for estimating enumeration and VWM capacities, separate blocks of trials were run in which the saliency of one item was manipulated. One item was changed, with respect to the other items, by either increasing its bottom-up or top-down saliency (Figure 2). In the former case, the visual contrast with the background and/or the size of the Gabor was increased. Manipulations of stimulus luminance and size have been shown to increase attention to that item even when these manipulations are task irrelevant [37]. Top-down saliency/priority was manipulated by adding a memory-guided saccade task. In these blocks of trials, a red dot was presented, along with the fixation point, at the beginning of the trials and participants were instructed to memorize this location in order to make a saccade there once the central fixation point was removed. A dim grey point (10% contrast) was present at that location, after the fixation point was removed, in order to allow participants to check their saccadic accuracy. In counting trials, the fixation point disappeared after the mask was removed, while in the VWM task it disappeared after the test probe was removed.

The experiment was run on a PC, using the Psychophysics toolbox [41], [42] and MATLAB (Mathworks, Inc.). Stimuli were displayed on a Mitsubishi Diamond Pro 2070 monitor at 75 Hz refresh rate. The display was viewed from a distance of 80 cm.

#### Eye tracking

The position of the right eye was monitored using the Eyelink 1000 video-based tracker. Eye position was calibrated at the beginning of each session and fixation correction was run before each block of trials. Trials were excluded in which eye-tracking was lost or participants failed to make the saccade to the remembered location within 500 ms.

#### Analysis

A sigmoid function provided a good fit to the percent correct data distribution for the different number of items in the enumeration task. We thus took, for each subject, the flex of the sigmoid curve as an estimate of the subitizing range [7]. Percent correct in the VWM task was determined by percent correct in same/different orientation judgments. Cowan's K was calculated based on the number of items (N), proportion of hits (H) and false alarms (FA) according to the formula *k* = N(H – FA) [9].

### Results and Discussion

**Figure 3:**
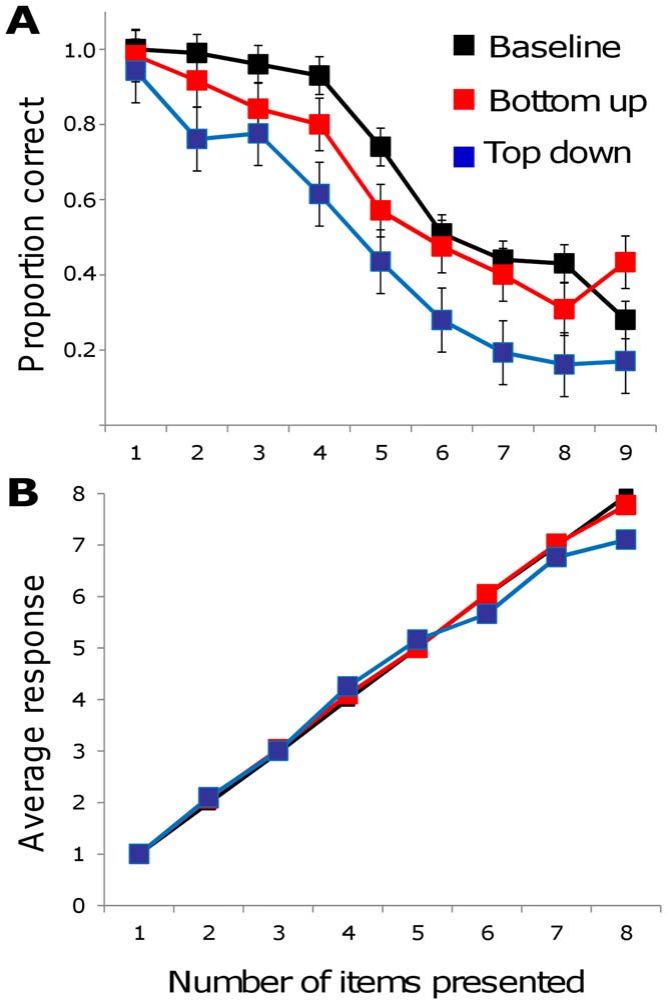
Influence of saliency manipulations on enumeration performance. A. Across all three conditions (shown as separate symbols and lines), percent correct enumeration decreased as a function of the number of items; F(8,5) = 37.93, p<0.001). Bars represent one standard error. B. Analysis of participant responses as a function of the actual number of items presented. Although performance was worse on trials with unequal salience, the most common response remained similar across the various conditions.

As expected, enumeration performance was near perfect for up to 3–4 items in the baseline condition (Fig. 3A, black line and symbols). When one item had a higher contrast and size compared to the background, however, performance dropped, particularly when there were three or more items (Figure 3A, red symbols) (main effect of bottom-up saliency condition: F(1,5) = 7.953, *P*<0.05). Likewise, manipulating top-down, task-related saliency by displaying an item at the location of a memorized saccade target led to an even stronger decrease in performance (Figure 3A, blue symbols) (main effect of top-down saliency condition: F(1,5) = 18.685, *P*<0.01). Considering both bottom-up and top-down saliency compared to bottom-up saliency, there was a main effect of salience condition (F(2,4) = 21.61, *P*<0.01) and no interaction between saliency condition and numerosity (F(2,4) = 1.17, N.S.).

The effect of top-down saliency was stronger, resulting in worse performance in the top-down condition compared to the bottom-up saliency condition (F(1,5) = 11.01, *P*<0.01). The interaction between saliency condition (top-down or bottom-up) and numerosity was not significant, suggesting that both types of saliency influenced performance in a qualitatively similar fashion (F(1,5) = 1.86, N.S.). In the case of the top-down attention task, the effect of saliency was measurable already with two-item displays (t(5) = 2.89, *P*<0.05) as well as three-item displays (t(5) = 2.80, *P*<0.05). In contrast, the effect of bottom-up saliency was seen with three-item displays (t(5) = 2.67, *P*<0.05) but less so with two-item displays (t(5) = 2.09, *P* = 0.091).

The saliency manipulation decreased the subitizing range, as measured by the flex in the sigmoid curve which was fit to percent correct for each number of items for each participant (baseline: mean *r* ^2^ = 0.87; bottom-up: mean *r* ^2^ = .85; top-down, mean *r* ^2^ = .94). Compared to the control condition (estimate of 5.97), this range was reduced by both bottom-up (estimate of 5.11; t = 2.57, *P*<.05) and top-down (estimate of 5.23; t = 2.77, *P*<0.05) saliency manipulations.

It is important to note that performance remained unchanged when there was only one stimulus. The saliency manipulation did not influence percentage correct in the one-item displays. This fact makes it unlikely that the effect was caused by a general, non-specific reduction in performance.

It is interesting to note that the presence of the highly salient item did not lead to a consistent over- or under-estimation of numerosity (Fig. 3B). One hypothesis, if subitizing results from a limited number of “slots” or “pointers” would be that a highly salient item might take more slots/pointers, leading to an over-estimation (if two slots were used for the salient item) or, conversely, could lead to under-estimation if items need to reach a threshold activation in order to attract a pointer. On the other hand, our finding could also be predicted if reducing the overall resources for the non-salient item results in less precision for the other items. The current findings do not allow us to discriminate between these possibilities.

**Figure 4:**
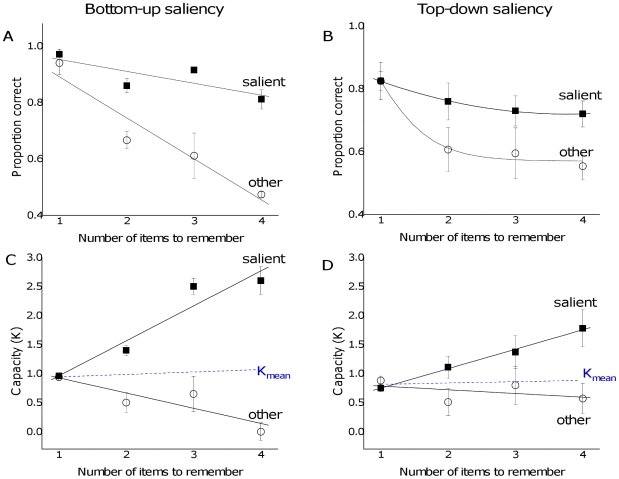
Influence of saliency on proportion correct and capacity in the VWM task. The left column shows trials in which the salient item was defined by contrast while the right column shows trials where one item was displayed at a task-relevant location. The bottom row shows data from the top row re-plotted in terms of capacity (Cowan's K). Dotted lines show the estimated capacity across the entire set of items (including both salient and non-salient items). Bars represent one standard error of the mean.

As with enumeration, visual working memory capacity was also influenced by changes in the relative saliency of the items. Memory for the most salient item remained high, independent of increased set size, while performance for the non-salient item dropped precipitously with set size (Fig. 4A) [main effect of saliency: F(1,5) = 7.95, *P*<0.05]. A similar trend was found in trials in which one item was more salient because it was presented at the saccade target location (Fig. 4B) [main effect of saliency: F(1,5) = 18.68, *P*<.01]. Thus, both bottom-up and top-down saliency influenced VWM in similar ways.

The saliency manipulation decreased the overall capacity estimate (Cowan's K [9]). Capacity decreased from around 1.53 items in the baseline condition to 0.89 items in the bottom-up [t = 3.67, *P*<.02] and 0.79 items in the top-down conditions [t = 3.90, *P*<.02]. This finding is consistent with our hypothesis that a highly salient item could reduce overall VWM capacity to only one single item. In fact, the VWM capacity appeared to be focused on the single most salient item: the estimated value of K for the salient item continued to increase up to 4 items, but dropped dramatically for the non-salient items (Fig. 4, panels C and D). While the trend is similar for both top-down and bottom-up trials, it is particularly evident in the bottom-up contrast manipulation (Fig. 4C) in which the estimated capacity for non-salient items in the four item trials was reduced to zero. When the overall capacity for all items was averaged across all of the items in the display, it remained constant as a function of set size (Fig. 4C,D: dotted line). This suggests that the presence of the salient item reduced overall capacity but that this influence was independent of set size.

**Figure 5:**
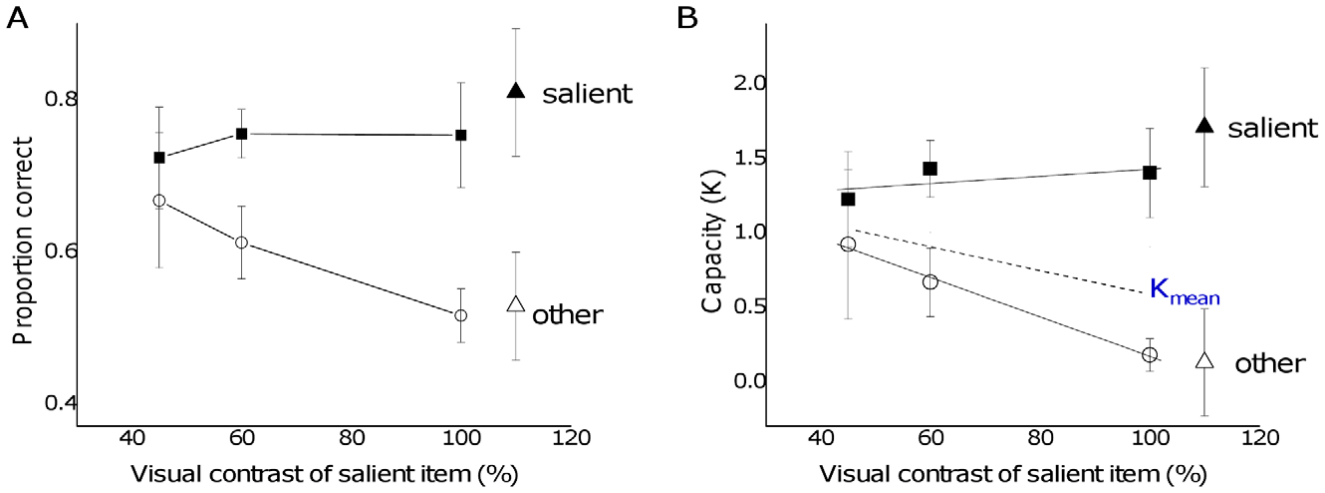
The influence of bottom-up saliency on proportion correct and capacity in the VWM task. The number of items (set size) was held constant at three. A. Proportion correct for salient and non-salient items as a function of visual contrast of the salient item, including trials in which the stimulus was also larger in size (triangles). B. Data from panel A re-plotted to show the capacity estimate (Cowan's K). The dotted line shows how the estimated capacity of the entire set of items (salient and non-salient) decreases when one item becomes increasingly more salient than the others. Error bars show standard error of the mean.

Next we measured the influence of bottom-up saliency by keeping the number of items constant. The influence of the salient item on performance increased as a function of its difference in contrast with the other items, with the largest effect when both the contrast and the size of the salient item were increased (Fig. 5, triangles). In terms of proportion correct (Fig. 5A), memory for the salient item remained relatively constant while performance for the non-salient items decreased to chance as the relative salience difference increased. This trend is also shown clearly in the capacity estimates (Fig. 5B), where the capacity for the salient item remained high while capacity for non-salient items dropped to zero. Again, the overall capacity was determined by saliency, dropping dramatically as the difference in contrast between the salient and non-salient items was increased (Fig. 5B, dotted line).

Overall, the pattern of results was consistent with the hypothesis that capacity is influenced by the relative saliency of the items. As predicted, overall capacity decreased when the relative salience difference between items was large. These effects were similar in both tasks (enumeration and VWM) and for both saliency manipulation (bottom-up and top-down).

## Experiment 2: Influence of Saliency on Working Memory Capacity for Oriented Bars

Numerous studies have shown that capacity estimates are reduced for VWM involving more complex stimuli [10], and are particularly low for grating stimuli compared to bars [43]. Consistent with this finding, the K estimates for VWM for the Gabor stimuli in the first experiment were around only 1.5 items, which is on the low end of capacity estimates in change-detection tasks. Previous work suggests that memory capacity for simple oriented bars should be higher than for oriented gratings [43]. Thus, we tested whether the pattern of results found in the first experiment would be replicated with different subjects and stimuli in a second experiment using oriented bars rather than Gabor grating stimuli.

### Methods

#### Subjects

Ten adult subjects took part in this experiment for course credit. Informed consent was obtained for all participants. None of the participants in the second experiment had participated in the first experiment.

#### Stimuli

The black bars were displayed within a 1.2 degree of visual angle window, in one of sixteen possible locations arranged around the central fixation point. In the salient condition, one of the bars was approximately 10% larger in size and was colored either white or red, randomized across trials.

#### Procedure

**Figure 6:**
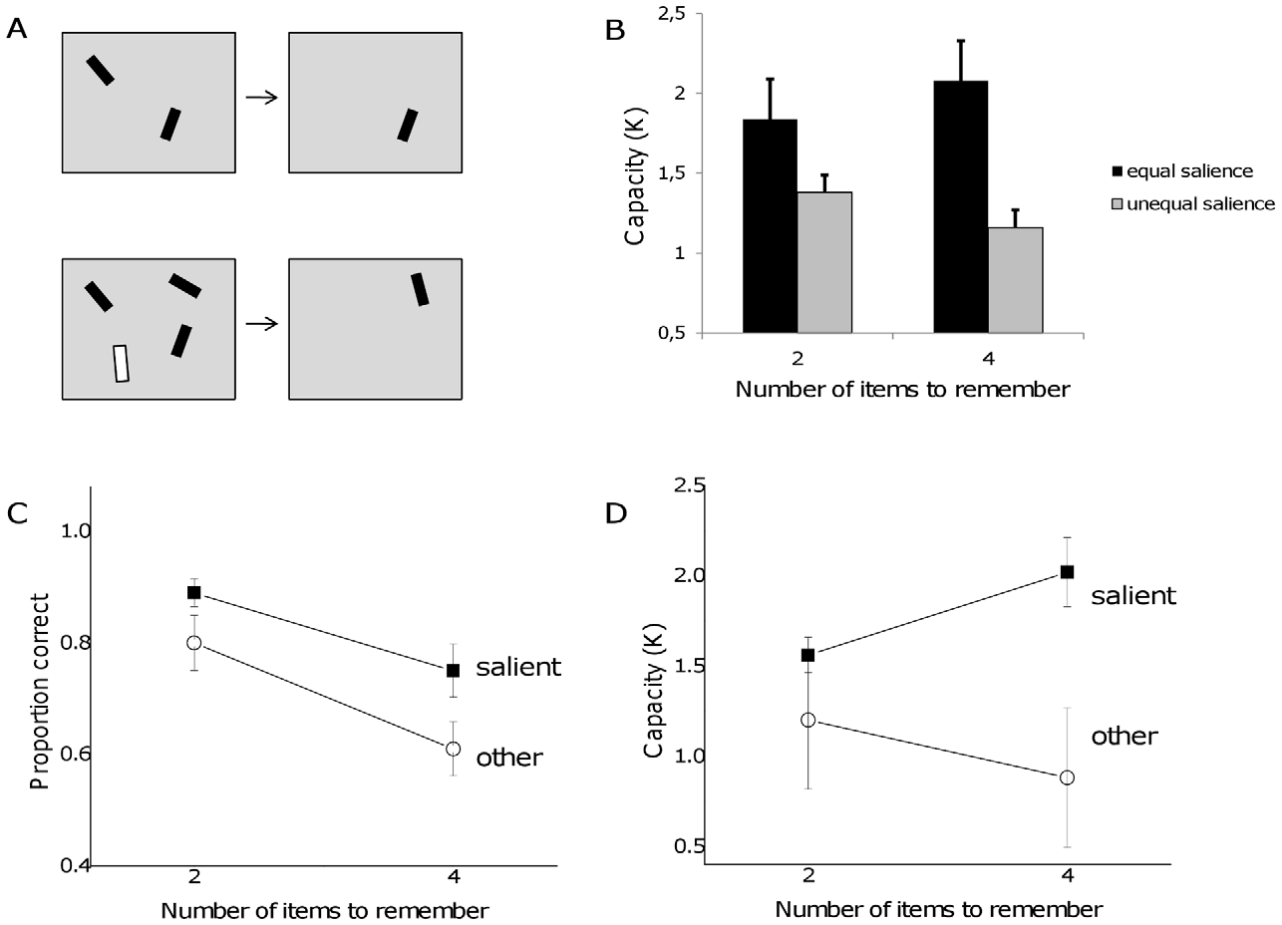
Test of the saliency manipulation on visual working memory for oriented bar stimuli. A. Participants first viewed a memory set of two or four items. On half of the trials, one of the bars was larger and a different (white or red) color. The test stimulus was presented either in the same orientation or rotated by 45 degrees clockwise or counter-clockwise. B. Capacity was larger on trials with equal salience. C. Performance correct was higher for salient items than the non-salient items, on trials with unequal salience. D. The capacity estimate was lower for the non-salient stimuli.

The procedure in the visual working memory task was identical to that of the first experiment, with the exception of using oriented bars rather than Gabor patches (Figure 6A). On each trial, the set size of the memory display was either 2 or 4 items, and one test stimulus was shown. The test stimulus was always presented as black and of normal size (rather than 10% bigger than the other items), even on trials in which the stimulus in that position had been a “salient” stimulus, in order to make sure that any differences were not a result of improved visibility of the test stimulus. The test stimulus was either identical or was rotated 45 degrees clockwise or counter-clockwise from the original orientation. Overall, there were 20 trials (10 same and 10 changed orientation trials) for each set size for the three conditions: equal salience, unequal salience with a change to the salient item, unequal salience with a change to the non-salient item. The 120 trials were run in a single block.

### Results and Discussion

Performance was best on trials in which all of the bars had the same color (Figure 6B). The capacity for trials with equal salience was estimated at around 2 items, which was larger than that found in the previous experiment, consistent with a previous report [43]. The saliency manipulation decreased the overall capacity estimate for the trials with unequal salience, F(1,9) = 8.98, p = 0.015. On those trials, performance was better for the salient stimulus compared to the non-salient stimulus, F(1,9) = 16.94, p = 0.003 (Figure 6C,D). Performance was worse for four items compared to two, confirming previous findings of limited capacity for orientation discrimination, F(1,9) = 16.94, p = .003.

The pattern of results replicate those found in the first experiment, showing again that unequal saliency among items decreases overall capacity. Thus, the results of the first experiment were not specific to a particular stimulus type or set of participants. The overall capacity was larger for oriented bars compared to Gabor stimuli, as previously reported [43]. Nonetheless, the highly salient stimulus dominated visual memory.

## Experiment 3: Integration of Bottom-Up and Top-Down Saliency

In the third experiment, we pitted bottom-up and top-down saliency against each other in the VWM task. In each display, there was one item with higher contrast (bottom-up salient item), one item at the saccade target (top-down salient item) and one baseline (non-salient) item. If all forms of salience are combined in a master map [17], then the influences of top-down and bottom-up saliency can be compared. In other words, the top-down manipulation of making one location relevant for a saccadic eye movement should be equal to a certain quantifiable boost in bottom-up contrast (perhaps, for example, a 50% or 100% increase in salience). On the other hand, if our proposal is incorrect, then the effects of different attention/salience manipulations might be unrelated or orthogonal to each other.

### Methods

#### Subjects

The six subjects from the first experiment participated in this study.

#### Procedure

The trials procedure was identical to the top-down (saccade task) condition in Experiment 1. However, in addition to the saccade task manipulation, there was also an item whose contrast and size was manipulated, as in the bottom-up salience condition in Experiment 1. Thus, on each trial, there was both a bottom-up and top-down salient item.

### Results and Discussion

**Figure 7:**
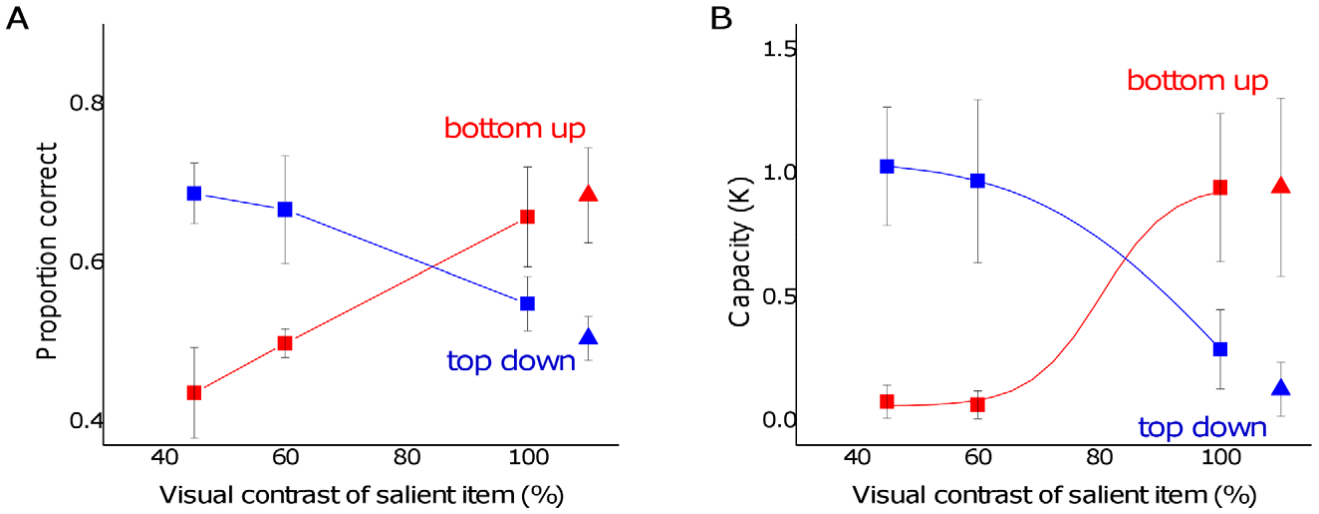
Performance on trials in which both top-down and bottom-up saliency were manipulated, showing (A) proportion correct and (B) estimated capacity. Bars represent one standard error.

The item displayed at the saccadic target dominated memory when the relative bottom-up visual salience difference was small (Fig. 7, blue symbols). With larger boosts to the bottom-up visual salience of an item, however, it captured memory completely (Fig. 7, red symbols), at which point there was chance performance for the top-down salient item. As shown in Figure 7B, the overall capacity remained around one item, which is consistent with the first experiment and our hypothesis that large differences in relative salience lead to small capacity estimates.

The overall pattern of results is consistent with the hypothesis that bottom-up and top-down saliency feed into a single “master map” of attentional priority and saliency (MAPS). These two sources of salience appeared to compete with each other, such that only about one item wins the competition to be individuated and remembered.

Consistent with our hypothesis, bottom-up and top-down saliency information competed to drive memory performance. The influence of task-relevant factors were critical when the visual contrast difference (our measure of bottom-up saliency) was relatively small, but eventually the bottom-up difference was able to overpower the task relevance, capturing the entire visual working memory capacity. This finding is consistent with the claim that all aspects of attentional priority are integrated into a single “currency” of saliency [17].

## Experiment 4: Comparing Individuation and VWM Capacity

**Figure 8:**
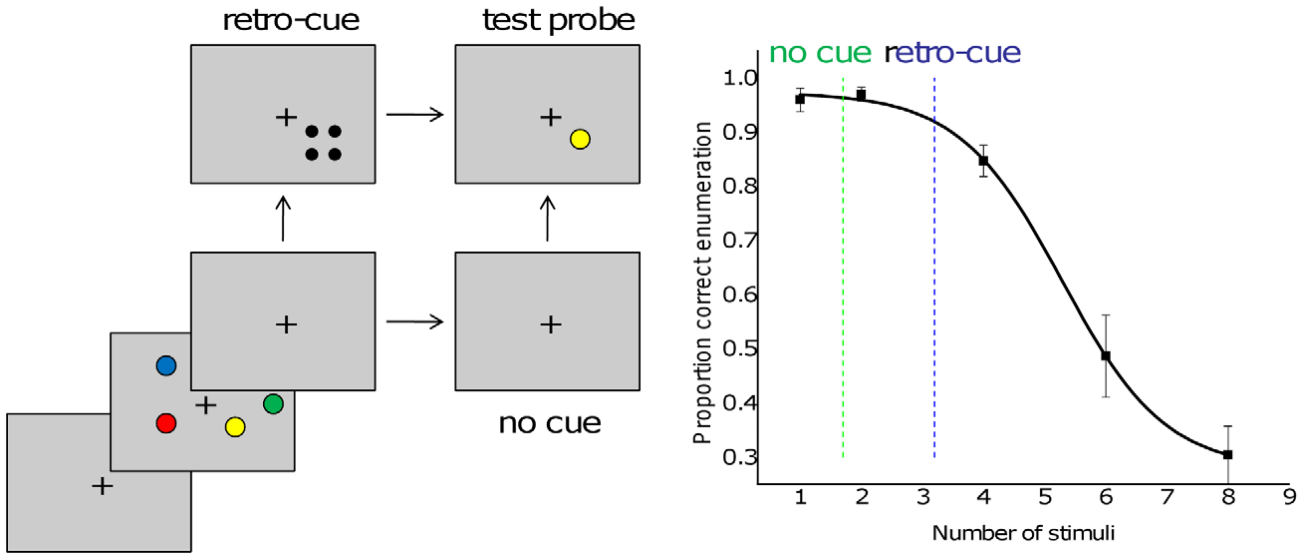
Visual working memory capacity examined using a retro-cue procedure. A. The color change working memory test. On half of the trials, there was a retro-cue presented 200 ms after the disappearance of the memory set. B. Proportion correct in the enumeration task. The vertical lines show estimated capacity based on the normal (no cue) and the retro-cue trials. The capacity estimate in the retro-cue trials better matches the point of inflection in the enumeration curve.

Our working hypothesis is that enumeration and VWM share an initial individuation stage. We have recently reported that individual differences in capacity limits for enumeration and visual working memory were strongly correlated [14]. However, the particular VWM task we used tended to give a small capacity estimate and small individual differences between participants. Average subitizing range about double that of VWM capacity for orientation in our first experiment. This finding might have resulted from the particular VWM task we used. Although enumeration and VWM might share the individuation stage, the second stage of identifying and representing orientation and keeping that information linked to a spatial location over time might have added further limitations in our VWM tasks. In contrast, a VWM task which reduces the role of maintenance and uses simple stimuli should increase the similarity between subitizing range and VWM span. To this end, we made two changes to the method used in Experiment 2. First, we introduced a retro-cue on some trials (Figure 8A). The retro-cue paradigm involves displaying a spatial cue shortly after the disappearance of the memory set that indicates which item will be tested [36]. Interestingly, this simple manipulation can increase capacity estimates by a factor of two compared to the more typical method used in visual working memory studies such as the first three experiments reported here. In addition, we hypothesized that replacing the orientation memory test with a simpler color category change detection task should reduce the degree to which precise visual information, possibly involving areas outside of the saliency maps in posterior parietal cortex, was needed to solve the task [34]. Thus, we hypothesized that the simpler VWM task with less maintenance requirement should lead to similar capacity in the two tasks.

### Methods

#### Participants

Thirteen adult subjects took part in this experiment for course credit. Informed consent was obtained for all participants. None of the participants had participated in the other experiments.

#### Stimuli

The memory set contained two, four or eight colored disks (Figure 8A) subtending approximately 1.5 degrees of visual angle, located within a four by four grid of possible locations within the central 10 degrees of the screen. The retro-cue, shown on 50% of trials, was made up of four small dots placed at the corners of the location where one of the colored disks had been presented. There were 9 possible colors (red, green, black, white, blue, yellow, brown, purple, orange). In the enumeration task, the targets to count were identical to those in the first experiment.

#### Procedure

For the memory task, the procedure was similar to that of experiment 2: a memory set was shown for 200 ms, followed by a 2 second delay, then a test probe item was shown at the same spatial location as one of the test items. The probe was either the same color as in the memory set or was changed to one of the remaining colors that had not been used in that memory set. On half of the trials, the retro-cue was shown 200 ms after the offset of the memory set and stayed visible until the test item was shown (Figure 8A). Participants reported, using a keypress, whether the item was the same or different color as in the memory set. In the enumeration task, a set of items (1, 2, 4, 6 or 8) were presented briefly as described previously for the first experiment.

### Results and Discussion

With the retro-cue, participants had an estimated capacity of more than 3 items, while on normal trials (no retro-cue) the estimated capacity was less than 2 items. In the enumeration task, participants were perfect for one or two items, then showed a drop in performance for the 4 item displays (Figure 8B). The pattern of results from both the enumeration and the retro-cue conditions were consistent with a capacity of approximately 3 items in both tasks. Moreover, in the case of the retro cue the individual differences in the two tasks were correlated. Participants who had a larger estimate of VWM capacity in the retro cue task (Cowan's K based on performance with 4 items) also had a higher estimate for subitizing range (Pearson's r = 0,762, p = 0.002). In contrast, there was no correlation between K and subitizing range without the retro cue (r =  −0.03, N.S.), perhaps due to the compressed estimates of VWM capacity without the retro cue).

## Discussion

When multiple items are simultaneously present in a display, they compete for our attention. This competition between items to reach higher levels of cognition may help to explain the fact that there are basic capacity limits in individuation and memory. Building on the idea of attention priority maps, we explored the possibility that competition between items might lead to similar effects on enumeration and visual working memory tasks. We modulated this competition by introducing differences in top-down or bottom-up saliency between the items. Consistent with our hypothesis, we found that that increasing the relative salience of one item decreased the overall individuation capacity as measured in two different tasks, enumeration and working memory. In the working memory task, the influence of saliency and set size were independent: the relative salience of the items determined overall capacity, which remained constant across set size. This pattern of results was similar for bottom-up and top-down salience, consistent with the hypothesis that there is a *Map of Attentional Priority and Saliency* (MAPS) that integrates sensory, motor and task relevance into a single neural “currency” [17].

In contrast to our manipulation of *relative* salience, some previous studies of enumeration had manipulated *general* salience [44], [45]. For example, connecting dots together (which might reduce the salience of individual items) led to underestimation of items for displays of 9 – 15 dots [44]. Palomares and Egeth [45] tested the influence of contrast on enumeration of gratings inside the subitizing range (1–4) items and beyond (up to 10 items). They reported that the general finding of similar performance of set sizes up to four remained even with low contrast stimuli. Reducing general salience of all items might influence the data-limited aspect of capacity limits (lower signal to noise ratio), while changing relative salience leads to conflict between items due resource-limited processes [46]. Our results add to previous studies of general salience by showing an effect of competition between items that might be missed if all items are equally salient. Moreover, our results provide new predictions regarding manipulations of general salience.

Overall, our results are consistent with the hypothesis that a spatial map limiting rapid individuation underlies performance in both the VWM and enumeration tasks. Although limited capacity in these two tasks has previously been studied separately in the domains of memory and numerical cognition, respectively, our findings suggest that both tasks rely on a common stage of individuation that is not itself task specific [14]. Our results are consistent with suggestions that many cognitive tasks “recycle” basic and multi-purpose sensorimotor capacities [16], [47]. Thus, even limits in complex tasks with abstract concepts, such as numbers, may be grounded in fundamental perceptual abilities.

In contrast to the predictions of fixed capacity (“slot”) models [4], [8], we found that the number of items that could be enumerated or remembered was not fixed, but varied based on the relative salience of the items. Inconsistent with some resource models, however, we found that set size manipulations did not change estimated capacity. Also, we found that the “resource” was not equally divided among the items, but was dominated by one, or at most a few, complete items. The finding that a salient item would dominate the other items would not have been directly predicted by either capacity or resource models, but instead fits in well with theories suggesting that limited capacity results from competition between items [29], [31], [48]. While a few studies of VWM have examined the role of stimulus factors in determining capacity, this involved changing the complexity of all of the items simultaneously. We found that making one item more salient causes it to dominate other items, reducing the overall capacity, and suggesting that competitive interactions between items determines access to the saliency map underlying individuation.

Given the demonstrated importance of competition in selective attention, our results can be considered within the context of winner-take-all models of saliency maps [31]. To date, however, most computational models have focused on finding a single winner such as in a visual search task. Indeed, it is important for a saliency map to be able to yield a single “winner” in order to guide actions such as a saccadic eye movement or pointing. However, as described above, many real-life tasks require individuating multiple items at once. There must also be flexibility in order to individuate multiple items when there is more than one target in a sequence of actions. This is particularly the case in rapid enumeration, where we need to quickly estimate the number of “peaks” in the saliency map rather than deduce the single most salient item. However, the need to converge on a single item, when needed, provides a different perspective on capacity limits. Unlike previous models, which start by trying to explain the upper limit on capacity (ie. “5 +- 2”), winner-take-all models emphasize the need to be able to converge on a single item for actions such as pointing, grasping or aiming at a target [49]. By this alternative logic, capacity should be viewed as 1 + N items, with N varying based on task and individual differences.

The experiments reported here provide additional evidence for a shared resource in both enumeration and VWM tasks (see also [13], [14]). However, the second stage in our model also plays an important role in determining capacity, as confirmed in the fourth (retro-cue) experiment (see [50] for recent evidence that VWM shares resources with other visual tasks). For VWM, it is clear that the additional factor of cognitive control [51], which is captured well by resource models, is needed to explain why performance is worse for remembering highly complex objects compared to simple ones. For example, one might individuate three different complex shapes, but then fail to maintain the detail of all three shapes in memory due to problems with cognitive control. Individuation is, we suggest, necessary but not sufficient for VWM, and so it creates the upper limit on VWM capacity. There is growing evidence that VWM involves sustaining a link between spatial location (encoded in the location maps) and visual properties which are encoded in feature-sensitive areas in visual cortex [26]. Any interference during the delay period which disrupts the location maps, such as a second individuation task [14] or TMS over posterior parietal cortex [52], [53] can dramatically reduce VWM performance. Thus, we suggest that MAPS might be involved throughout the visual working memory task, during the encoding and maintenance of the items.

The finding that attention was involved in subitizing agrees with recent studies that have questioned the idea of “pre-attentive” subitizing mechanisms [54]–[56]. In those studies, a second task was added which reduced the accuracy of enumeration even in the subitizing range. In the Vetter et al. [56] study, for example, participants performed a central foveal task while trying to enumerate items in the periphery. Their overall pattern of results looked very similar to ours (see: [56], Figure 3). Their results fit well with the MAPS framework if one views their central target (in the secondary task) as highly salient due to both its increased bottom-up salience (it is at the fovea) and its task relevance. Indeed, as their task became more difficult at the central location (presumably making it necessary to make it increasingly an attentional priority) performance fell even further. Our experiments are novel, however, in that they show that *relative salience*, not just attentional allocation *per se*, plays a role in determining the subitizing range.

### Conclusions

In sum, our results suggest that capacity limits in enumeration or working memory are not actually unique phenomena constrained to a single cognitive domain. The overall findings are consistent with the idea that attentional priority and saliency maps may be the critical bottleneck in the process of individuation. Our findings suggest that it is the competition between items that leads to capacity limits, such that any imbalance in salience between items can lead to a “winner-take-all” situation in which otherwise relevant items are effectively blocked from access to the brain regions that guide our actions, thoughts and memories. We suggest that the non-salient items simply do not “count” in the initial saliency map [17], and thus are not able to be rapidly individuated. Even relatively modest differences in bottom-up or top-down salience can render items irrelevant and virtually invisible to the individuation mechanisms, as part of the necessary process of dynamically allocating neural resources to the most important items in a complex scene.
